# Lifelong Exercise as a Modulator of CD4 ^+^ T Cell Immunometabolism

**DOI:** 10.1111/eci.70204

**Published:** 2026-05-05

**Authors:** Bruna Spolador de Alencar Silva, Amanda Veiga Sardeli, Ricardo R. Agostinete, Helena Batatinha, Alessandra Peres, José César Rosa‐Neto, José Teixeira, Manuel J. Coelho‐e‐Silva, Paulo J. Oliveira, Fábio Santos Lira

**Affiliations:** ^1^ Department of Physical Education, Exercise and Immunometabolism Research Group, Postgraduation Program in Movement Sciences Sao Paulo State University (UNESP) Presidente Prudente Brazil; ^2^ Department of Inflammation and Ageing University of Birmingham Birmingham UK; ^3^ Laboratory of Investigation in Exercise (LIVE), Department of Physical Education Sao Paulo State University (UNESP) Presidente Prudente Brazil; ^4^ University of North Carolina at Greensboro Greensboro North Carolina USA; ^5^ Rehabilitation Sciences Graduate Program, Universidade Federal de Ciências da Saúde de Porto Alegre (UFCSPA) Porto Alegre Brazil; ^6^ Department of Cell and Developmental Biology University of São Paulo São Paulo Brazil; ^7^ CNC‐UC, Center for Neuroscience and Cell Biology, University of Coimbra Coimbra Portugal; ^8^ CiBB, Center for Innovative Biomedicine and Biotechnology, University of Coimbra Coimbra Portugal; ^9^ University of Coimbra, FCDEF Coimbra Portugal; ^10^ University of Lisbon, CIPER Cruz Quebrada Portugal

**Keywords:** Aging, Energy metabolism, Exercise, Immune function, Immunosenescence

## Abstract

**Background:**

It is commonly assumed that aging and chronic low‐grade inflammation compromise adaptive immunity, particularly the function and metabolism of CD4^+^ T cells. The preceding are key regulators of immune responses. These immunological alterations contribute to increased susceptibility to infections, diminished vaccine efficacy and the progression of age‐related diseases. In contrast, adolescence and young adulthood tend to be characterized by more robust immune responses, though these are heavily influenced by modifiable lifestyle factors such as habitual physical activity, level of cardiorespiratory fitness, diet and body adiposity. Emerging evidence suggests that sustained physical activity throughout life may preserve CD4^+^ T cell competence by favourably modulating their metabolic programming.

**Methods:**

The current narrative review explores how lifelong physical exercise impacts CD4^+^ T cell metabolism, with particular emphasis on the developmental window of adolescence and the long‐term benefits of early and sustained physical training across the lifespan. Molecular mechanisms linking exercise to metabolic reprogramming of T cells were summarised in parallel with attenuation of immunosenescence and inflammation over the lifespan.

**Results:**

This review suggests that lifelong exercise may reprogram CD4^+^ T cell metabolism, enhancing oxidative phosphorylation at rest and glycolytic control upon activation, thereby improving Th17/Treg balance, reducing chronic inflammation and enabling effective effector T cell responses. In this context, exercise initiated early in life may act as a critical modulator by promoting optimal immune function from childhood and establishing a functional peak that helps preserve immune competence during aging.

**Conclusions:**

Lifelong and early‐life exercise may reprogram CD4^+^ T cell metabolism, strengthening immune balance and preserving immune function during aging.

## Graphical abstract

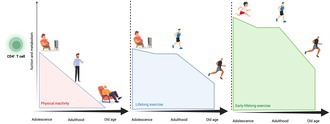

## 1 Introduction

Contemporary lifestyle has increased the prevalence of physical inactivity and sedentary behaviour in the worldwide population, favouring obesity, metabolic diseases and low‐grade inflammation. The latter involves a mild, although consistent increase in the levels of pro‐inflammatory markers like interleukin 6 (IL‐6) and tumour necrosis factor‐alpha (TNF‐α) from children to adult age [1, 2]. Over time, the lack of physical activity and sedentary behaviour contributes to chronic low‐grade inflammation and may accelerate immunosenescence, thereby increasing susceptibility to chronic and infectious diseases, particularly in older adults [3].

CD4^+^T cells play a central role in regulating immunity and their metabolic reprogramming is decisive to their differentiation (i.e.,: from T CD4^+^ th0 to T CD4^+^ th1, th2, th17, or regulatory T (Treg) profiles) and fate [4, 5]. CD4^+^ T cells, also known as helper T cells, originate from haematopoietic stem cells in the bone marrow and mature in the thymus. Upon antigen recognition presented by MHC‐II molecules on antigen‐presenting cells, naïve CD4^+^ T cells become activated and differentiate into distinct functional subsets. This differentiation is shaped by cytokine signals, antigenic stimulation and metabolic cues that collectively tailor immune responses to pathogens and tissue microenvironments [6]. Regular practice of physical activity efficiently prevents CD4^+^ T cell senescence [7, 8], favouring an efficient immune response, a robust immunosurveillance (e.g.,: in the context of cancer), reducing chronic inflammation and the risk of autoimmune diseases [9].

In this context, cellular metabolism refers to the dynamic coordination of bioenergetic and biosynthetic pathways that sustain immune cell activation and function. Beyond merely providing energy, these metabolic routes determine T cell fate by coupling nutrient availability to signalling networks such as AMP‐activated protein kinase–mechanistic target of rapamycin (AMPK–mTOR) and hypoxia‐inducible factor‐1 alpha (HIF‐1α). For instance, naïve CD4^+^ T cells predominantly rely on oxidative phosphorylation (OXPHOS) and fatty acid oxidation (FAO) to preserve quiescence and longevity. Upon activation, a metabolic switch toward aerobic glycolysis supports rapid proliferation and effector cytokine synthesis, favouring Th1 and Th17 differentiation. Conversely, the maintenance of mitochondrial oxidative metabolism and lipid utilization underpins regulatory T cell (Treg) stability and anti‐inflammatory activity. Therefore, metabolism acts as an intrinsic determinant of CD4^+^ T cell plasticity and functional specialization [10, 11].

Given the essential role of metabolism in immune cell differentiation and the established influence of physical activity on systemic physiology [12, 13], one attractive hypothesis is that continuous maintenance of regular exercise throughout life (from adolescence to older ages) might contribute to the long‐term programming of T cell metabolism, particularly in CD4^+^ subsets. This metabolic programming could involve a more efficient basal oxidative profile, characterized by reduced glycolytic activity at rest, coupled with a greater capacity to upregulate glycolysis upon activation. Such flexibility may reflect enhanced mitochondrial fitness and metabolic plasticity, ultimately promoting an anti‐inflammatory phenotype and more effective immune responses when required.

**FIGURE 1:**
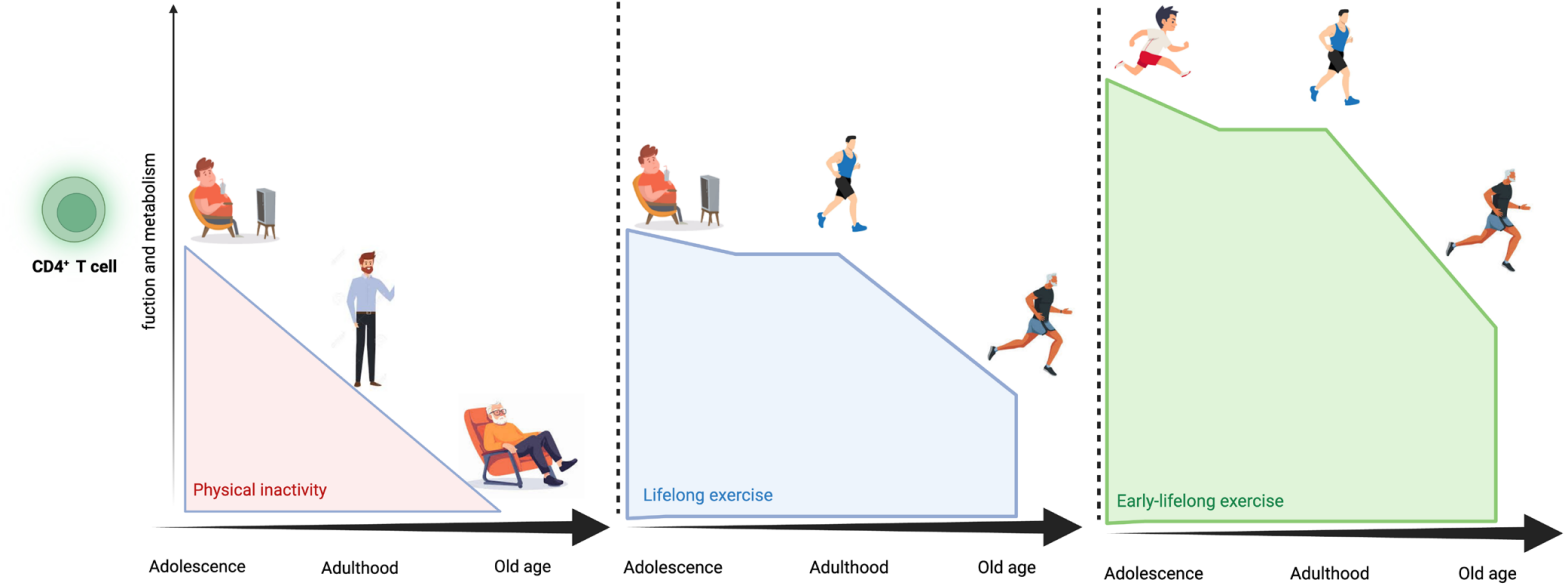
Hypothetical framework illustrating how physical exercise initiated early in life acts as a critical immunomodulatory factor by promoting optimal immune function from childhood and establishing a better “immunometabolic functional peak”. This early advantage may confer sustained benefits to CD4^+^ T cell metabolism and immune competence across aging, compared with exercise initiation in adulthood.

This review aims to explore whether initiating exercise early in life, for example during adolescence, confers greater benefits to immune function compared to starting in adulthood or older age, taking into account the cumulative years of physical activity. It also discusses potential molecular mechanisms through which physical activity regulates energy metabolism and CD4^+^T cell function (Figure 1).

## 2 Low‐Grade Chronic Inflammation Throughout Life

Low‐grade chronic inflammation refers to a mild but persistent increase in circulating pro‐inflammatory cytokines over an extended period [2]. This condition can emerge at different life stages and is shaped by behavioural, psychosocial and physiological factors.

During adolescence, chronic low‐grade inflammation is particularly sensitive to environmental and lifestyle determinants. Diets rich in processed foods and sugars are associated with increased adiposity and inflammatory markers, whereas healthier dietary patterns rich in fruits, vegetables and whole grains exert protective effects [14, 15]. Adverse socioeconomic conditions can promote unhealthy behaviours and are linked to higher inflammatory levels [16]. In addition, poor sleep quality, insufficient sleep duration and psychosocial stressors such as family conflict or academic pressure contribute to immune dysregulation [17, 18, 19]. Conversely, social support and positive parenting act as protective factors, reducing inflammation and improving health outcomes [20, 21].

In adulthood, low‐grade inflammation is strongly associated with metabolic disturbances, particularly those related to excess adipose tissue [22]. Increased fat mass correlates with a sustained pro‐inflammatory profile that contributes to insulin resistance, cardiometabolic disorders and non‐alcoholic fatty liver disease [23, 24, 25]. Excess adiposity can also accelerate immunosenescence, as obese adults exhibit immune dysfunction resembling that observed in aging, characterized by reduced immune responsiveness and persistent inflammation [26, 27]. Our group demonstrated increased expression of the exhaustion marker programmed cell death protein 1 (PD‐1) in CD4^+^ T cells from sedentary obese young adults, concomitant with hyperleptinemia, insulin resistance and dyslipidemia [28]. PD‐1 signalling suppresses T cell proliferation and cytokine production and is associated with metabolic reprogramming marked by increased oxidative metabolism and reduced glycolysis [29, 30].

In older adults, chronic low‐grade inflammation, termed inflammaging, is highly prevalent and contributes to increased morbidity and mortality from chronic diseases [31, 32, 33]. Lifelong inflammatory exposure and immunosenescence interact bidirectionally, as chronic inflammation accelerates immune cell aging, while senescent immune cells perpetuate inflammation, forming a self‐sustaining cycle [32, 33].

Collectively, these findings demonstrate that low‐grade inflammation is shaped by a continuum of biological and environmental factors acting throughout life, from adolescence to aging. Although its multifactorial origins are well documented, the specific effects of persistent inflammation on adaptive immunity, particularly CD4^+^ T cell metabolism and function, remain insufficiently explored. Emerging evidence indicates that chronic low‐grade inflammation progressively impairs CD4^+^ T cell activation, differentiation and maintenance, undermining the capacity to mount effective immune responses and maintain homeostasis across the lifespan [34, 35, 36]. Understanding how chronic inflammation reshapes CD4^+^ T cell bioenergetics is essential for elucidating mechanisms underlying immune decline and highlights regular physical exercise as a potential modulator of immune health [37, 38].

In the following sections, we discuss how CD4^+^ T cell function and metabolism influence health and disease across life stages and examine the impact of physical (in)activity on immune regulation from adolescence to aging.

## 3 Function and Metabolism of CD4 ^+^ T Cells: Implications for Health and Disease

Beyond their classical immune role, CD4^+^ T cells contribute to systemic metabolic homeostasis by modulating tissue inflammation, insulin sensitivity and energy metabolism, particularly in adipose tissue and the gastrointestinal tract [39, 40]. Their marked metabolic plasticity during activation and differentiation positions CD4^+^ T cells at the core of the immunometabolic network relevant to obesity, type 2 diabetes and metabolic syndrome.

Cellular metabolism is essential for T cell activation, differentiation and effector function [41, 42]. Immune cells depend on substrates such as glucose, fatty acids, amino acids and glutamine to sustain proliferation, survival and cytokine production [43]. Metabolic pathways influence immune outcomes beyond ATP generation by regulating intracellular signalling, epigenetic remodelling and transcriptional programs [44]. Upon activation, CD4^+^ T cells markedly increase glycolysis, even under normoxic conditions, a phenomenon known as the Warburg effect, which is normally associated with cancer cells [45]. Failure to undergo this metabolic reprogramming compromises IL‐2 and IFN‐γ production, impairing clonal expansion and adaptive immunity.

Naïve T cells rely primarily on OXPHOS, regulated by catabolic pathways such as AMPK. In contrast, activated CD4^+^ T cells increase glycolysis, fatty acid synthesis and glutaminolysis to support rapid proliferation and effector differentiation, largely mediated by mTOR signalling [46]. Cytokine production is tightly linked to metabolic state, with IL‐2 being highly glycolysis‐dependent, whereas IFN‐γ can be supported by both glycolysis and OXPHOS [47]. In chronic inflammatory and nutrient‐restricted environments, impaired coordination between these pathways contributes to functional exhaustion.

After antigen clearance, most effector cells undergo apoptosis, while a subset differentiates into memory T cells. During this transition, reduced inflammatory signalling leads to AMPK‐ and MAPK‐mediated inhibition of mTOR and activation of autophagy, which is essential for memory CD4^+^ T cell formation and longevity [48]. Memory T cells predominantly rely on FAO and OXPHOS and exhibit distinct mitochondrial morphology [49].

Throughout life, physiological and environmental factors shape immunometabolic pathways. Aging profoundly impacts CD4^+^ T cells, leading to immunosenescence characterized by thymic involution, a decline in naïve T cells, an increased memory‐to‐naïve ratio and reduced responsiveness to novel antigens [50]. Additional hallmarks include telomere shortening, impaired proliferation and helper function and increased Th17 polarization [51, 52]. Persistent infections, such as cytomegalovirus, further drive effector T cells into senescence [53]. The accumulation of senescent/exhausted T cells (CD28^−^CD57^+^KLRG1^+^PD‐1^+^) promotes chronic low‐grade inflammation through resistance to apoptosis and secretion of senescence‐associated inflammatory factors [54].

T cell differentiation, exhaustion and aging are tightly regulated by the metabolism–epigenetics axis [55]. Terminally differentiated effector memory T cells (TEMRA) are more prevalent in older adults and display altered mitochondrial function, including increased mitochondrial mass but reduced membrane potential and impaired mitophagy [56]. Aged naïve CD4^+^ T cells exhibit reduced glycolysis and defective one‐carbon metabolism, likely driven by mitochondrial dysfunction and impaired autophagy [57, 58]. In contrast, memory CD4^+^ T cells from older individuals have been reported to have increased respiratory capacity, accompanied by elevated ROS production and pro‐inflammatory cytokine secretion [59].

Aging also disrupts the Th17/Treg balance, favouring pro‐inflammatory Th17 cells and reduced Treg‐mediated regulation, thereby increasing susceptibility to infections and autoimmunity [52]. In parallel, glutaminolysis remains a critical metabolic pathway, with older CD4^+^ T cells relying more heavily on glutamine to sustain cytokine production and proliferation. Young CD4^+^ T cells can compensate via glycolysis and OXPHOS when other pathways are blocked. In contrast, inhibition of glutaminolysis (e.g., with 6‐diazo‐5‐oxo‐l‐norleucine (DON)) selectively blunts cytokine production and proliferation in old, but not young, CD4^+^ T cells, underscoring this age‐specific dependence [60].

Beyond natural aging as a major driver of CD4^+^ T‐cell metabolism and function, a premature immunosenescence phenotype can be influenced by behavioural and environmental factors. Sedentary behaviour and obesity, for example, promote early immunosenescence in young adults [28] and various modulators, including diet, gut microbiota and circadian rhythms, directly impact the epigenetic and functional programming of T cells throughout life [55]. In cardiometabolic diseases, the hypoxic and lipid‐rich adipose microenvironment promotes CD4^+^ T cell dysfunction, Treg depletion and sustained inflammation. Experimental models demonstrate that early‐life mitochondrial dysfunction is sufficient to induce profound metabolic reprogramming, pro‐inflammatory cytokine production and premature senescence in CD4^+^ T cells [61, 62].

These findings demonstrate that mitochondrial dysfunction, even at early ages, is sufficient to profoundly alter CD4^+^ T‐cell metabolism and function, predisposing to a pro‐inflammatory phenotype and accelerating immunosenescence. Therefore, identifying immunometabolic alterations already in youth is essential, given that intrinsic dysfunctions in CD4^+^ T cells contribute to chronic inflammation and accelerate systemic biological aging [9]. Taken together, these lines of evidence show that metabolic dysfunction in CD4^+^ T cells may lie at the centre of several pathological processes, influencing infections, autoimmunity, metabolic diseases and aging itself and represents a promising target for therapeutic interventions.

## 4 Impact of Physical (In)activity on the Immune System and Health Across the Lifespan

Modern society has undergone profound behavioural changes driven by technological development, urbanization and socioeconomic transitions. These changes have favoured sedentary habits, defined as activities performed in sitting or reclining positions with energy expenditure ≤ 1.5 metabolic equivalents (METs) and reduced overall physical activity, defined as any skeletal muscle movement requiring energy expenditure [63, 64]. Consequently, the global prevalence of overweight and obesity has tripled since 1975, reaching 43% overweight and 16% obese adults in 2022 [65]. Excess adiposity promotes the release of pro‐inflammatory adipokines, contributing to low‐grade systemic inflammation from early life onward [1, 2].

Regular physical activity, particularly structured exercise, counteracts these effects through multiple mechanisms [66]. In addition to reducing fat mass, exercise stimulates the release of myokines and induces an increase in plasma IL‐6 levels, which is subsequently followed by a coordinated anti‐inflammatory response characterized by elevated levels of IL‐1 receptor antagonist (IL‐1ra) and IL‐10. Concurrently, soluble TNF receptors increase, contributing to the suppression of TNF‐α activity. Altogether, this cascade reflects a systemic anti‐inflammatory effect induced by exercise [67, 68]. Furthermore, there is incipient evidence from a meta‐analytical study of 146 exercise‐controlled trials suggesting while the anti‐inflammatory effects of aerobic training are thight influenced by increased energy expenditure, associated with reductions in IL‐6, resistance and combined training might trigger anti‐inflammatory effects by anabolic mechanisms related to IGF‐1 increase [66].

Engagement in physical activity early in life may also induce epigenetic adaptations influencing immune cell differentiation and metabolic flexibility, thereby reducing susceptibility to chronic diseases later in life [69, 70]. In fact, a study in male mice showed exercise training starting from 1‐month‐old controls cytokine response and mitigates sepsis when exposed to lipopolysaccharide challenge even after 11 months of detraining [71]. These persistent benefits were linked to epigenetic modifications affecting immunometabolic pathways, including changes in mTOR signalling and metabolites involved in immune regulation.

Adolescence represents a critical period for establishing lifelong health behaviours. Despite this, 81% of adolescents aged 11–17 years do not meet the WHO recommendation of ≥ 60 min/day of moderate‐to‐vigorous physical activity [72]. This widespread inactivity coincides with rising rates of obesity and metabolic dysfunction, increasing the risk of early immune dysregulation [14, 15]. Adolescents with obesity exhibit alterations in T cell profiles, characterized by a reduction in total T cells (CD3^+^) and in regulatory and anti‐inflammatory subsets (CD4^+^CD28^+^, IL‐4^+^ and FOXP3^+^), alongside an increased frequency of pro‐inflammatory CD4^+^IL‐17A^+^ cells, as well as lower circulating IL‐10 levels [73]. This profile suggests a shift toward a pro‐inflammatory immune state, likely driven by excess adipose tissue, which promotes Th17 expansion while impairing regulatory mechanisms, thereby contributing to systemic inflammation and metabolic dysfunction associated with obesity. Puberty is also characterized by a peak in lymphoid tissue development, during which the immune system may display enhanced capacity to respond to infections. This phase is accompanied by thymic enlargement, supporting the production and maturation of T lymphocytes (CD4^+^ and CD8^+^), as well as immunoglobulin synthesis, particularly IgG [74]. Nevertheless, physical inactivity during this critical developmental period has been associated with elevated inflammatory markers and alterations in T cell phenotypes. Collectively, these findings suggest that pediatric obesity and sedentary behaviour may accelerate features of immunosenescence, potentially predisposing individuals to chronic diseases and impaired immune competence later in life [75, 76].

In adulthood, insufficient physical activity remains highly prevalent, with 27.5% of adults failing to meet minimum activity recommendations [65]. Persistent inactivity contributes to the growing burden of non‐communicable diseases and shifts healthcare systems toward treatment rather than prevention. Conversely, physically active adults exhibit improved immune surveillance, including higher CD4^+^ T cell counts, lower inflammatory profiles and enhanced mucosal immunity [77, 78]. Strengthening of the mucosal barrier may be partly attributed to increased levels of secretory immunoglobulin A (SIgA) associated with physical activity, which acts as a first line of defence against pathogens [79]. CD4^+^ T cells are key orchestrators, regulators and direct effectors of the immune response, enhancing the activity and memory of other immune cells while contributing to pathogen clearance [80, 81]. The increased CD4^+^ T cell levels associated with exercise suggest that regular physical activity strengthens these immune functions [82]. In this context, regular exercise also improves vaccine‐induced immune responses by enhancing the functional capacity of CD4^+^ T cells. Exercise can stimulate antigen‐specific CD4^+^ T cell expansion and increase cytokine production following vaccination, thereby potentiating vaccine effectiveness [83]. In addition, exercise‐induced modulation of CD4^+^ T cell metabolism, favouring OXPHOS over glycolysis, supports efficient immune responses while limiting chronic inflammation [84, 85] Collectively, these adaptations contribute to more effective pathogen recognition, immune regulation and long‐term protection.

With aging, physical activity becomes increasingly important for mitigating immunosenescence and chronic low‐grade inflammation, which impair responses to infections and vaccination [86, 87]. Physical inactivity increases markedly after 60 years of age (~ 37%), exacerbating immune and metabolic dysregulation [65]. Mechanistically, aging is characterized by thymic involution, reducing naïve T cell output and favouring the accumulation of memory and senescent T cells [30, 88]. Concurrently, elevated IL‐6, TNF‐α and C‐reactive protein (CRP) levels perpetuate inflammaging, a chronic pro‐inflammatory state associated with frailty and multimorbidity [89]. Regular exercise helps preserve CD4^+^ T cell function, maintain mitochondrial health and enhance immune resilience. Long‐term exercise is also associated with a more “youthful” T cell profile in older adults, suggesting partial protection against thymic involution and immunosenescence [3, 90].

In summary, across the lifespan, physical activity is a key determinant of immune competence. Early‐life exercise shapes immune and metabolic programming, sustained activity in adulthood preserves immunosurveillance and lifelong engagement delays age‐associated immune decline, underscoring the importance of promoting physical activity from youth to old age.

## 5 Impact of Acute and Chronic Exercise on CD4 ^+^ T Cell Immunometabolism

The metabolic demands during acute exercise can change CD4^+^ T cell identity, which could impact the metabolites disposal and promote epigenetic reprogramming [91]. In line with this, serum collected from healthy young adults after an acute endurance exercise bout marginally altered specific parameters of CD4^+^ T cell metabolism, indicating sensitivity of these cells to exercise‐induced changes in circulating cytokines and substrates [84].

Acute high‐intensity exercise may also transiently influence CD4^+^ T cell phenotype and function. For instance, an exhaustive aerobic protocol increased immunosuppressive CD4^+^CD25^−^CD39^+^ T cells when peripheral blood mononuclear cells (PBMCs) from obese men were incubated with autologous post‐exercise serum [92]. High‐intensity interval exercise further modulates CD39 and CD73 expression on CD4^+^ T cells, potentially contributing to anti‐inflammatory effects through purinergic signalling [84].

To date, the only study directly evaluating acute exercise effects on CD4^+^ T cell metabolism is Gebhardt et al. (2024) [93]. Using a crossover design involving downhill and level running, the authors demonstrated that a single bout of moderate‐intensity aerobic exercise did not induce robust transcriptomic or metabolomic reprogramming in CD4^+^ T cells assessed ex vivo with autologous post‐exercise serum. However, individuals with higher VO_2_max exhibited greater mitochondrial capacity and a metabolomic profile oriented toward oxidative phosphorylation, indicating that baseline cardiorespiratory fitness modulates CD4^+^ T cell metabolic responsiveness.

Chronic physical exercise not only modulates T cell numbers but also significantly enhances their functional capacity. Six‐month exercise program consisted of resistance and aerobic training increased proportion of activated CD4^+^ T cells, along with the restoration of their proliferative capacity in breast cancer patients [94]. Eight weeks of combined aerobic and resisted exercise were able to reduce intracellular concentrations of IL‐8 and to increase IL‐13 and TNF‐α in CD4+ T lymphocytes in subjects with stable COPD [95]. Moreover, growing evidence indicates that chronic exercise training enhances the functional fitness of CD4^+^ T cells. For instance, a 10‐week supervised program performed alternating high‐intensity and low‐intensity intervals induced improvements in oxidative metabolism of CD4^+^ T cells in individuals with rheumatoid arthritis, with paralleling gains in cardiorespiratory fitness [96].

Beyond glucose and lipid metabolism, glutamine metabolism is tightly linked to exercise‐induced immune adaptations. Both acute and chronic exercise modulate plasma glutamine availability and lymphocyte glutamine utilization. Intervention studies combining exercise training with L‐glutamine supplementation have demonstrated shifts in naïve, effector and activated CD4^+^ T cell subsets in young athletes and older adults [97, 98]. Chronic exercise stabilizes systemic glutamine levels, reduces low‐grade inflammation, enhances mitochondrial efficiency and promotes metabolic flexibility in lymphocytes, supporting glutaminolysis as a key pathway for CD4^+^ T cell proliferation and effector function, contributing to the anti‐inflammatory and immunomodulatory effects associated with regular physical training [99].

Across life stages, regular exercise training or sustained moderate‐to‐high physical activity levels support immune homeostasis [3, 100] by preserving thymic output, increasing naïve T cell frequency, preventing telomere attrition, maintaining the balance of pro‐ and anti‐inflammatory cytokines and delaying immunosenescence [3, 85, 101]. High cardiorespiratory fitness levels (≥ 3000 m·min^−1^·week^−1^) have also been associated with protection against latent viral reactivation, possibly via enhanced mobilization and function of virus‐specific T cells [91, 102].

Chronic exercise further modulates the Th17/Treg axis. Chronic high‐intensity exercise has been shown to modulate and enhance Treg number and function [103]. Lifelong exercise, as observed in master athletes capable of cycling 100 km in < 6.5 h (men) or 60 km in < 5.5 h (women), is associated with reduced Th17 frequencies compared with age‐matched non‐athletes [7]. Similarly, 6–8 weeks of structured exercise training increases Treg frequency while reducing Th17 cells in populations with inflammatory diseases [104, 105]. Mechanistically, increased Treg‐mediated IL‐2 consumption and a larger naïve CD4^+^ T cell pool may contribute to this shift [7, 106]. Importantly, Treg frequency should be interpreted in light of functional capacity, particularly in aging, where compensatory Treg expansion may coexist with reduced suppressive activity. Therefore, it is fundamental to associate the effects of exercise and aging on T cell phenotype with the functionality of these T cells.

Exercise training also counteracts mitochondrial aging in immune cells [107]. Hodgman et al. recently reviewed exercise training effects in PBMCs metabolism [90]. Multiple training modalities increase oxygen consumption rate and OXPHOS capacity in PBMCs, changes associated with improved skeletal muscle respiration [108], lower fatigue scores [109] and increased VO_2_max [110]. Repeated bouts of exercise promote lymphocyte trafficking through peripheral tissues, followed by homing to lymphoid organs, contributing to immune surveillance [111]. We speculate that this migration process potentially exposes T cells to myokines and other exercise‐derived signals that favour long‐term metabolic reprogramming.

Lifelong exercise likely promotes cyclical activation of AMPK during exercise and mTOR during recovery, supporting metabolic efficiency and functional resilience in CD4^+^ T cells. This dynamic regulation supports metabolic programming aimed at enhancing cellular efficiency and function. The repeated activation of these pathways may underlie the beneficial adaptations observed in T cells over time. Collectively, lifelong exercise is proposed to induce metabolic remodelling in T cells, positively influencing not only their frequency but also their functional capacity. Consistently, physically active older adults exhibit greater mitochondrial dependence and lower glucose reliance in CD4^+^ T cell subsets, whereas inactive peers show higher energetic demand that could be particularly associated with the hyperinflammatory responses and potentially higher frequency of senescent cells in older adults with lower levels of physical activity [112].

Growing evidence suggests that enhanced metabolic efficiency in immune cells is a key mechanism through which lifelong exercise preserves adaptive immunity during aging [22, 113]. For example, a 30‐min steady‐state cycling protocol performed at 15% above the individual lactate threshold significantly influenced CD4^+^ T cell metabolism and differentiation, promoting polarization toward either Th17 or Treg lineages depending on the physiological context [91]. Clinical studies further support these findings; a 12‐week Tai Chi Chuan intervention reduced glycated haemoglobin levels and increased Treg counts in individuals with type 2 diabetes, with improvements in glycemic control correlating with enhanced FoxP3 expression in T cells [114, 115]. Similarly, aerobic exercise programs, such as a 4‐week treadmill protocol at 65% HRmax, improved the Th17/Treg balance in autoimmune conditions, including multiple sclerosis and psoriasis, in parallel with clinical improvement [116, 117]. Lifelong exercise has also been associated with lower circulating IL‐6 and higher IL‐15 levels, cytokines closely linked to metabolic regulation and immune function [7].

Nevertheless, further evidence is needed to establish a causal link between physical exercise, immunosenescence and overall health. Exercise‐induced energy metabolism programming of immune cells represents a promising target for future research. In regards to different types of exercise, although most of the information provided in this review comes from aerobic exercise interventions, there is also some evidence that resistance training [118, 119, 120, 121, 122] can increase CD4^+^ T cell frequencies, reduce CD4^+^ CD25^+^ T cells, CD4^+^ PD‐1^+^ T cells and senescent‐like T cells, although there is still limited information about their metabolism and functional activity [123]. A summary of the studies included in this section can be seen in Table 1.

**TABLE 1:** Summary of studies from the section “Impact of Acute and Chronic Exercise on CD4^+^ T cell immunometabolism”.

Ref	Study	Type	Population	Sample Size (*n*)	Exercise details	Control group	Main CD4 ^+^ T cells findings
**Acute exercise**							
[91]	Spielmann et al., 2016	Acute aerobic	Healthy adults	10	30‐min cycling (at a power output corresponding to 15% above their individual lactate threshold (BLT))	Within‐subject	Altered CD4^+^ identity
[84]	Dorneles et al., 2019	Acute aerobic HIIE	Healthy adults (high physical fitness vs. low hysical fitness)	30	10 × 60s HIIE (85% HRmax intercepted by 75 s of recovery at 50% Hrmax)	Within‐subject	CD39/CD73 modulation
[92]	Dorneles et al., 2020	Acute aerobic	Obese adults	9	10 × 60s HIIE (85%–90% MAV intercepted by 75 s of active recovery (50% MAV)) vs. exhaustive exercise (stepping up and down from a step). The stepping rhythm was paced acoustically at 60 beats per min with the same periods (1 s) for stepping up and down, respectively.	Crossover	Post‐HIIE serum: ↓H4 acetylation, ↓NF‐κB, ↓TNF‐α, ↓IL‐6, ↑IL‐10, ↑CD4^+^CTLA‐4^+^, ↑CD4^+^CD25^+^CD39^+^/CD73^+^; Post‐exhaustive serum: ↑H4 acetylation, ↑mitochondrial depolarization, ↑TNF‐α, ↓CD4^+^CD25^+^CD73^+^, ↑CD4^+^CD25⁻CD39^+^; Both: ↑CD4^+^PD‐1^+^ and ↑CD8^+^PD‐1^+^.
[93]	Gebhardt et al., 2024	Acute aerobic	Healthy adults	15	45 min 70% VO_2_max (downhill run and a level run)	Crossover	↑CRF → ↑OXPHOS (CD4^+^ T cells)
[102]	Kunz et al., 2018	Acute aerobic exercise	Healthy adults	14	30‐min steady‐state cycling protocol at a power output 10%–15% above the power output at the individual BLT.	Within‐subject	↑ Virus‐specific CD4^+^
[97]	Krzywkowski et al., 2001	Acute aerobic exercise	Healthy adults (athletes)	10	2 h cycling at 75% VO_2_max (2 days) + oral glutamine or placebo during and up to 2 h post‐exercise.	Crossover	Exercise ↑ CD8^+^/CD4^+^ (CD28^−^/CD95^−^) & ↑ CD4^+^ memory (CD45RA^−^) (naive ↔) → 2 h post ↓ most lymphocytes; glutamine ↔ lymphocyte responses (↓ glutamine drop; ↓ neutrocytosis slight, no clinical effect).
[101]	Minuzzi et al., 2018	Acute aerobic exercise	Older and young adults	29	VO_2_max test → cycling start 75 W ↑ +25 W/3 min → fatigue	Yes	Master athletes → ↓ senescent CD8^+^ (naïve/CM/EM) & ↓ senescent CD4^+^ (naïve/EM); age ↑ SLEC CD8^+^ ↓ naïve CD8^+^; VO_2_max ↑ naïve CD4^+^ ↔ ↓ lymphocytes; ↔ CD4^+^/CD8^+^ subsets, EMRA & gene expression (CCR7, Fas‐L).
**Chronic exercise**							
[94]	Hutnick et al., 2005	Combined	Breast cancer	49	6 months resistance training and aerobic activity at 60%–75% functional capacity; 3× week	Yes	↑ Activated CD4^+^ and proliferating CD4^+^ CD69^+^ T cells
[95]	Uzeloto et al., 2022	Combined	COPD	23	8 weeks training (aerobic at 80% of the 6MWT and resisted with 60% of 1RM, both with progressive protocols based on the perception of dyspnea)	Yes	Cytokine modulation (intracellular ↓IL‐8 ↑IL‐13, ↑TNF‐α in CD4^+^ T cells)
[96]	Andonian et al.,2022	HIIT	Rheumatoid arthritis	12	10 weeks 10 alternating high‐intensity (80%–90% heart rate reserve) and low‐intensity (50%–60% heart rate reserve) intervals (60–90 s each).	Yes	HIIT → ↑CRF (RA) ↔ ↑CD4^+^ T cell basal & maximal respiration; ↔ ↑naïve CD4^+^ (CCR7^+^CD45RA^+^), ↑muscle CrAT activity, ↑FAO gene expression & acylcarnitines (parallel T cell–muscle oxidative metabolism adaptations).
[104]	Ito et al., 2025	Combined	COPD	50	8 weeks aerobic: Heart rate at 50%–80% of maximum (Karvonen: HR = HRrest + [(HRmax − HRrest) × 50%]), with intensity increased by 5% every two sessions or if Borg score < 4; Resistance: Three sets of 8 repetitions at 50% of 1RM, progressing to 3 × 12 reps; once 12 reps are achieved, increase load by 10% and reset to 3 × 8 reps.	Yes	Exercise training ↑ Tregs (total/activated) ↓ Th17 → Th17/Treg ↔ ↑ 6MWT, ↑ strength (upper/lower), ↑ daily physical activity.
[113, 114]	Yeh et al. [114]; [115]	Tai Chi	T2D	39	12 weeks: Tai Chi (37 forms) 3×/week, 60 min/session (10 min warm‐up, 40 min practice, 10 min cool‐down)	Yes	TCC ↑ CD4^+^CD25^+^ Tregs ↔ ↓ HbA1c; baseline DM2: ↓ T‐bet (GATA‐3/FoxP3 ↔) → after 12 weeks TCC: ↑ T‐bet.
[115]	Mähler et al. [116]	Aerobic	Multiple sclerosis	34	4 weeks; 3×/week, 1 h/session, Normoxic (NO) or hypoxic (HO) treadmill training	Yes	NO training → ↑ CD39^+^ Tregs ↓ CD31^+^ Tregs ↓ IL‐17A^+^ CD4^+^; HO → no immunological changes.
[105]	Papp et al. 2021	Combined	Healthy elderly with sedentary lifestyle	29	6‐week long functional conditioning gymnastic exercise program Aerobic low→40%–50% HRmax → 50%–60% (20 min) + TRX full‐body (3× 6–12 reps) → + Fitball balance 10 min; HR monitored.	No	Exercise → ↑ naïve Tc & HLA‐DR^+^ T cells, ↓ effector memory Tc → memory B rearranged; ↓ Tr1 (IL‐10) & ↓ CD4^+^CD127lo/−CD25bright Tregs (↔ improved body composition/performance).
[117]	Abd El‐Kader et al., 2018	Aerobic vs. Resistance	Sedentary elderly	80	Aerobic treadmill 40 min → HR‐based (220–age): 60%–70% HRmax (0–3 month) → 70%–80% (3–6 month). Resistance training 40 min → 9 machines, 3× 8–12 reps @60%–80% 1RM → load +5 lbs. after 3 × 8 reps for 3 consecutive days.	Yes	Aerobic & resistance → ↓ TNF‐α, IL‐6, CRP & ↑ IL‐10 (anti‐inflammatory; greater with aerobic) → ↑ CD3^+^, CD4^+^, CD8^+^ & ↓ CD4/CD8 (↑ effect in aerobic vs. resistance).
[118]	Lee et al., 2021	Resistance	Adults with ovarian cancer	12	12 weeks; 4×/week (Progressive resistance (4×/week) → 50–60 min session; 10 exercises; RM progression: 12RM (week 1–3) → 10RM (week 4–6) → 8RM (week 7–9) → 6RM (week 10–12))	Yes	EG vs. CG → EG ↑ Th1/Th2 → ↓ CD4^+^CD25^+^, CD4^+^PD‐1^+^, CD8^+^PD‐1^+^, CD8^+^TIGIT^+^ → ↑ CD4^+^/CD8^+^ sensitivity (↑ myokine/cytokine response).
[119]	Brito‐ Neto et al., 2019	Resistance	Adults with HIV	19	12‐week resistance (2×/week) → 3 sets × 8–10 reps (multi‐exercises) → intensity progressed to maintain RPE 7–8 (OMNI‐RES).	Yes	ER: ↑ CD4^+^T lymphocytes
[120]	Dinh et al., 2019	Strenght vs. Strenght ‐Resistance	Healthy elderly	100	6 weeks resistance training (2–3×/week) intensive strength training (IST): 3 × 10 @80% 1RM vs. strength‐endurance training (SET): 2 × 30 @40% 1RM vs. control (CON): flexibility (3 × 10–12 × 30 s).	Yes	SET → ↓ senescence‐prone T cells vs. COM
[121]	Ogbutor et al., 2022	Isometric handgrip exercise	Sedentary pre‐hypertensive adults	192	48 days maximum voluntary contraction test → handgrip 2 × 2 min @30% MVC (5 min rest) daily; after 24 day: EG1 stop, EG2 continue @30%, EG3 ↑ to 50% MVC (next 24 day).	Yes	EG2/EG3 → ↑ CD4^+^ & ↑ CD4/CD8, ↓ CD8^+^ → ↑ duration > ↑ intensity effect.
[98]	Monteiro et al., 2020	Combined	Elderly	84	4 weeks −60–75 min/session, 3×/week (alternate days); Aerobic 60%–75% HRmax (208–0.7 × age) + strength 50%–60% 1RM → Borg‐guided → 2–3×/week, 5–10 exercises/session. Groups: non‐practitioners (NP), and practitioners of combined‐exercise training (CET) were submitted to Influenza virus vaccination and supplemented with Gln (glutamine), groups: NP‐Gln, and CET‐Gln, or placebo NP‐PL and CET‐PL.	Yes	CET/NP + vaccine ± Gln → CET‐Gln ↑ IgM & IgA (NP‐Gln & CET‐PL ↑ IgM) → ↑ HI titers (CET‐Gln, NP‐PL, NP‐Gln) → ↑ naïve/effector CD4^+^ (NP‐Gln, CET‐Gln) & ↑ activated CD4^+^ (CET).
**Cross‐sectional study**							
[7]	Duggal et al., 2018	Fitness level	Older adults	125	Cyclists (maintained a high level of physical activity (cycling) for much of their adult live) vs. inactive	Yes	Cyclists vs. inactive elders: ↑ naïve CD4 T & RTE (≈ young), ↑ IL‐7 ↓ IL‐6 → ↓ immunosenescence (↓ Th17, ↑ Bregs).
[112]	Hazeldine et al., 2025	Fitness level	Healthy aduts/older	28	Higher vs. lower physically active	Yes	Older vs. young: ↑ MD ↓ GD; LPA ↑ protein synthesis & ↑ IL‐6^+^ T cells, HPA ↑ MD & no IL‐6↑ → PA improves metabolic flexibility & ↓ hyperinflammation.
[85]	Bartlett and Duggal, 2020	Fitness level	Healthy older adults	211	Physically active vs. sedentary group	Yes	Active vs. sedentary → ↑ naïve CD4^+^/CD8^+^ & ↓ memory CD4^+^/CD8^+^ → ↑ IL‐7 & IL‐15; IL‐15 ↔ ↑ naïve CD4^+^.

*Note:* ↔ = no change/no significant difference.

Abbreviations: COPD, chronic obstructive pulmonary disease; CRF, cardiorespiratory fitness; HIIE, high‐intensity intermittent exercise; HIIT, high‐intensity interval training; HR, heart rate; MAV, maximum velocity; T2D, type 2 diabetes; VO_2_max, volume of oxygen maximum.

**FIGURE 2:**
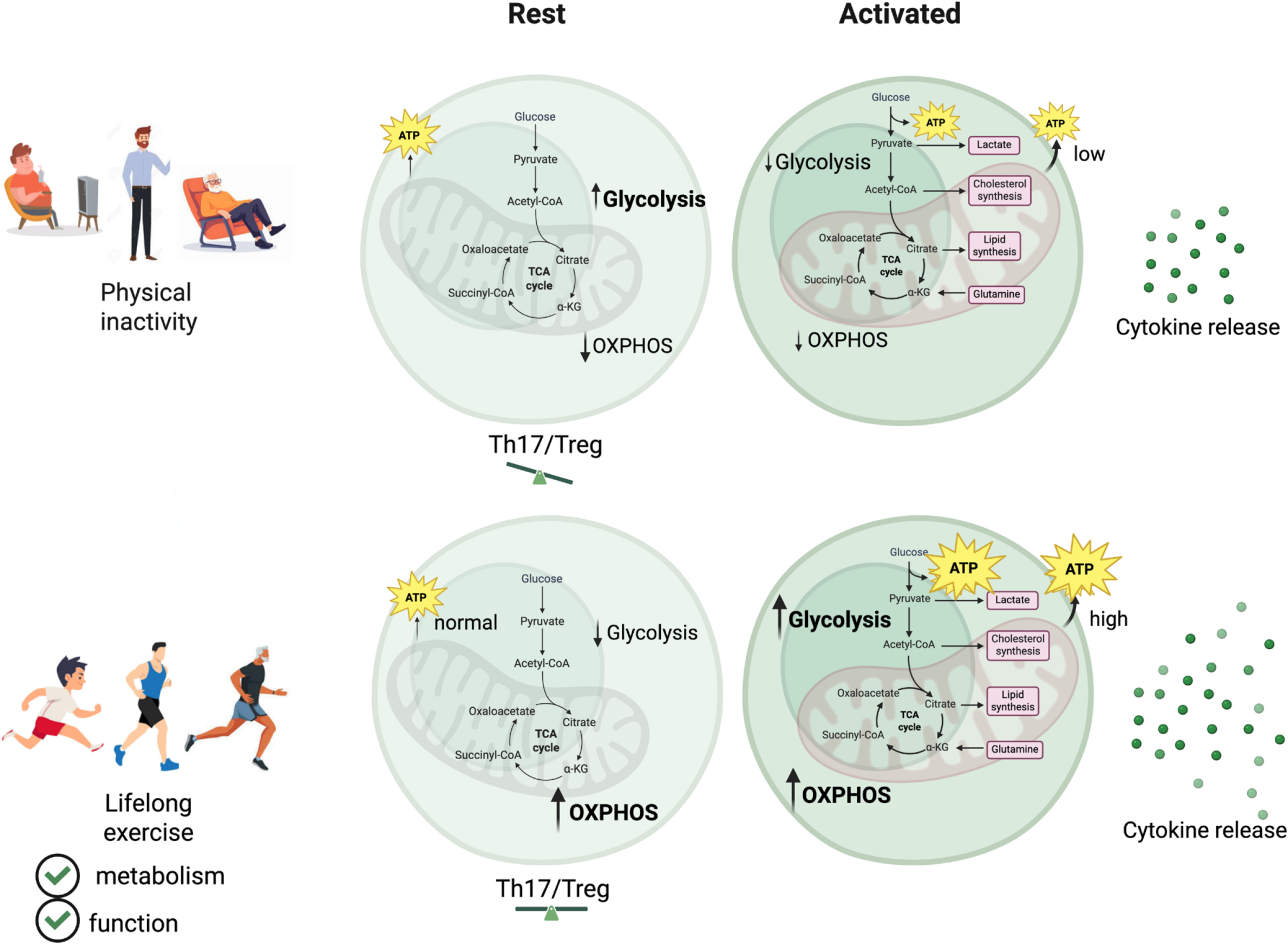
Proposed effects of lifelong exercise on CD4^+^ T cell mitochondrial function, glycolytic regulation, Th17/Treg balance and immune competence.

In this review, we highlight evidence from the current literature indicating that lifelong physical exercise has the potential to reprogram T cell metabolism, thereby preventing or attenuating the metabolic impairments typically observed in individuals with overweight/obesity and during the aging process. Specifically, we suggest that CD4^+^ T cells will show improved mitochondrial function to support oxidative phosphorylation at rest state and improved regulation of glycolysis upon activation, which will be associated with improved Th17/Treg ratio at resting condition. Therefore, this reprogramming may contribute to reduced chronic low‐grade inflammation and a more efficient activation of effector T cells when needed (Figure 2).

Based on the mechanisms discussed above, a potential intervention strategy to attenuate inflammaging would involve the early initiation and lifelong maintenance of regular physical exercise combined with lifestyle factors that support immunometabolic homeostasis [69, 70, 71]. Repeated exercise bouts activate metabolic regulators such as AMPK during exercise and mTOR during recovery, which may enhance mitochondrial function, oxidative phosphorylation capacity and metabolic flexibility in T cells over time, thereby supporting efficient immune activation while limiting chronic inflammatory signalling. At the cellular level, chronic training has been shown to improve oxidative metabolism of CD4^+^ T cells and modulate the Th17/Treg balance toward a more regulatory phenotype, which is associated with reduced inflammatory burden and improved immune regulation. A recent meta‐analytic compendium showed anti‐inflammatory effects of exercise are most pronounced with aerobic training, while resistance and combined training uniquely increase circulating IGF‐1 [66]. Higher training frequency and greater weekly training volume are associated with greater reductions in fat mass and insulin (both important for anti‐inflammatory effects), whereas exercising only twice per week appears insufficient to significantly improve several metabolic and inflammatory markers [66]. Exercise intensity alone does not consistently determine metabolic benefits, although higher intensities may enhance improvements in lipid profiles, also important to reduce chronic inflammation [66]. Complementary strategies, including balanced nutrition rich in anti‐inflammatory foods, adequate sleep and stress management, may further reinforce these adaptations by supporting mitochondrial function and nutrient‐sensing pathways involved in T cell differentiation. In fact, exercise training interventions significantly reducing fat mass, or improving glucose or lipid metabolism elicited greater anti‐inflammatory effects (variety of markers) in older adults and reduction in some markers, like TNF‐α and leptin only occurred with a parallel improvement in glucose metabolism [66]. Together, these combined behavioural interventions could promote long‐term immunometabolic resilience, delay immunosenescence and ultimately mitigate the development of inflammaging across the lifespan.

## 6 Future Perspectives/Opening Questions

### 6.1 What do We NEED to Know About Immunometabolism in CD4^+^ T Cells Through Life?

- Literature about Immunometabolism in CD4^+^ T cells during adolescence is scarce and most studies investigate middle‐aged and older adults. Alterations in the immunometabolic profile of CD4^+^ T cells with aging favour immunosenescence cells, leading to impaired immune function and low effectiveness against viruses and vaccination, mainly when aging is accompanied by increased adipose tissue depots.
- Physical exercise is associated with several favourable changes in immunometabolic profiles, such as a shift toward oxidative metabolism, enhanced anti‐inflammatory responses and improved vaccine responsiveness. However, these findings reflect correlations observed across different studies and it is not yet established whether exercise directly regulates these processes or how they interact mechanistically. Moreover, the duration, intensity and frequency of exercise required to achieve or sustain these potential benefits remain uncertain.

### 6.2 What do We NOT Know About Immunometabolism in CD4^+^ T Cells Through Life?

- The adolescence phase is a disturbing time from a physiological point of view, with many changes that are hormonal, behavioural and immunological. The monitoring of energetic metabolism in CD4^+^ T cells should be investigated in this stage. The understanding of the impact of sexual hormones over immunometabolism could explain the difference in immune response caused by sexual hormones.
- The scientific society needs to create or recreate ways to better understand the impact of lifelong exercise on immunometabolism changes and search for the implementation of strategies to reduce metabolic diseases.

## Author Contributions

B.S.A.S., A.V.S. and F.S.L. wrote the initial draft; B.S.A.S. prepared figures; B.S.A.S., A.V.S., F.S.L., R.R.A., H.B., A.P. and J.C.R.‐N. wrote manuscript; J.T., M.J.C.‐e.‐S. and P.J.O., critically reviewed the manuscript and made substantial contributions to the writing of the final version; and F.S.L. and P.J.O. provided funding.

## Funding

This work was supported by the National Council for Scientific and Technological Development (CNPq)—Brazil (Process number: 150760/2022–1) and the Coordination for the Improvement of Higher Education Personnel (CAPES), Brazil (code 001). F.S.L. and J.C.R.‐N. have granted research grants from the CCD/CMEPP FAPESP (Process 2025/07056–9). F.S.L. was granted a research scholarship (PQB) from the CNPq (303650/2024–9), and B.S.A.S. was granted a research scholarship from the São Paulo Research Foundation (FAPESP)/Brazil (Process Number: 2021/11932–8 and 2023/09216–8). R.R.A. was granted a research scholarship from PROPe UNESP (13/2022). A.P. has granted research grants from the FAPESP (Process 2025/23395–8). J.T. and P.J.O. are supported by the European Union, project PAS GRAS (Grant Agreement 101080329).

## Disclosure

The authors have nothing to report.

## Ethics Statement

The authors have nothing to report.

## Conflicts of Interest

The authors declare no conflicts of interest.

## Data Availability

Data sharing not applicable to this article as no datasets were generated or analysed during the current study.
